# Cholera Outbreak — Haiti, September 2022–January 2023

**DOI:** 10.15585/mmwr.mm7202a1

**Published:** 2023-01-13

**Authors:** Denisse Vega Ocasio, Stanley Juin, David Berendes, Kristen Heitzinger, Graeme Prentice-Mott, Anne Marie Desormeaux, Phaimyr D. Jn Charles, Jonas Rigodon, Valerie Pelletier, Reginald Jean Louis, John Vertefeuille, Jacques Boncy, Gerard Joseph, Valusnor Compère, Donald Lafontant, Lesly L. Andrecy, Edwige Michel, Katilla Pierre, Evenel Thermidor, David Fitter, Yoran Grant-Greene, Matthew Lozier, Samson Marseille, CDC Haiti Cholera Response Group, CDC Haiti Cholera Response Group, Ashley Andujar, Alondra Baez-Nieves, Lucy Breakwell, Sara Breese, Joan Brunkard, Mushtaq Dualeh, Thomas Handzel, Christine Lee, Andrea Martinsen, Taylor Osborne, David Shih, Amanda Tiffany, Maryann Turnsek

**Affiliations:** ^1^Division of Foodborne, Waterborne, and Environmental Diseases, National Center for Emerging and Zoonotic Infectious Diseases, CDC; ^2^Epidemic Intelligence Service, CDC; ^3^CDC Haiti Country Office; ^4^Global Immunization Division, Center for Global Health, CDC; ^5^National Public Health Laboratory, Ministère de la Santé Publique, Port-au-Prince, Haiti; ^6^Directorate of Epidemiology of Laboratory Research, Ministère de la Santé Publique, Port-au-Prince, Haiti; ^7^Direction Nationale de l'Eau Potable et de l'Assainissement, Port-au-Prince, Haiti.; CDC; CDC; CDC; CDC; CDC; CDC; CDC; CDC; CDC; CDC; CDC; CDC; CDC.

On September 30, 2022, after >3 years with no confirmed cholera cases (*1*), the Directorate of Epidemiology, Laboratories and Research (DELR) of the Haitian Ministry of Public Health and Population (Ministère de la Santé Publique et de la Population [MSPP]) was notified of two patients with acute, watery diarrhea in the metropolitan area of Port-au-Prince. Within 2 days, Haiti’s National Public Health Laboratory confirmed the bacterium *Vibrio cholerae* O1 in specimens from the two patients with suspected cholera infection, and an outbreak investigation began immediately. As of January 3, 2023, >20,000 suspected cholera cases had been reported throughout the country, and 79% of patients have been hospitalized. The moving 14-day case fatality ratio (CFR) was 3.0%. Cholera, which is transmitted through ingestion of water or food contaminated with fecal matter, can cause acute, severe, watery diarrhea that can rapidly lead to dehydration, shock, and death if not treated promptly (*2*). Haiti is currently facing ongoing worsening of gang violence, population displacement, social unrest, and insecurity, particularly in the metropolitan area of Port-au-Prince, including Belair, Bas-Delmas, Centre-Ville, Martissant, Cité Soleil, Croix-des Bouquets, and Tabarre, creating an environment that has facilitated the current resurgence of cholera (*3*). This report describes the initial investigation, ongoing outbreak, and public health response to cholera in Haiti. Cholera outbreak responses require a multipronged, multisectoral approach including surveillance; case management; access to safe water, sanitation, and hygiene (WASH) services; targeted oral cholera vaccine (OCV) campaigns; risk communication; and community engagement. This activity was reviewed by CDC and was conducted consistent with applicable federal law and CDC policy.*

## Epidemiologic Investigation

On September 30, 2022, DELR was alerted about a pediatric patient with acute watery diarrhea in Haiti’s Ouest Department,^†^ who was treated at a health center operated by Doctors Without Borders (Médecins Sans Frontières [MSF]). The patient was from the Carrefour Feuille area in Port-au-Prince, had been seen at an MSF health clinic on September 29, 2022, and died shortly after arrival. Also, on September 29, MSF reported a fatal case of acute, watery diarrhea from Cité Soleil, a densely populated commune of the metropolitan area of Port-au-Prince. On October 2, Haiti’s National Public Health Laboratory confirmed these two suspected cholera cases, both in the greater Port-au-Prince area, to be *V. cholerae* O1 (El Tor biotype) of the Ogawa serotype by culture and seroagglutination. Subsequent sequencing of one of these patients’ stool samples revealed the strains to be very similar to the strain that caused the cholera epidemic in Haiti in 2010, suggesting the resurgence of cholera in Haiti (*4*).

**FIGURE 1:**
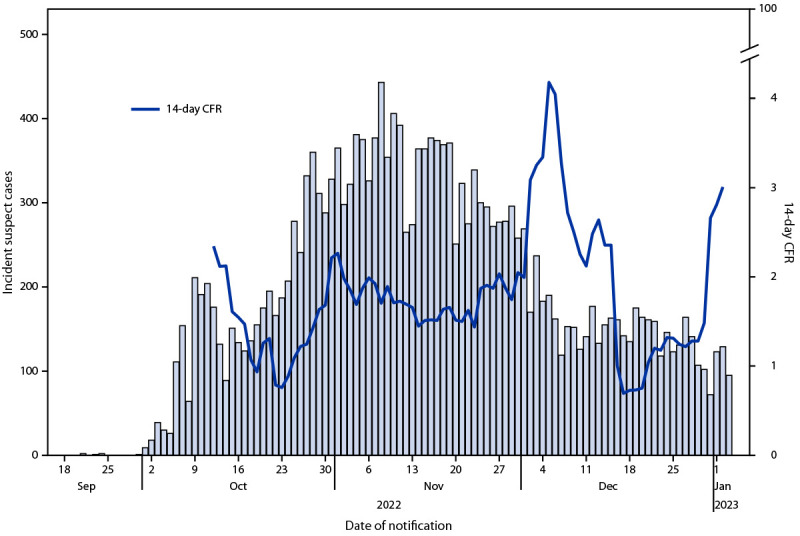
Date of notification of suspected cholera cases* and 14-day case fatality ratio^†^ — Haiti, September 2022–January 2023 **Abbreviation:** CFR = case fatality ratio. * From case-based surveillance reporting. ^†^ From alert-based reporting.

MSPP defined a suspected case of cholera as the occurrence of acute, watery diarrhea, with or without vomiting or dehydration, in a person of any age. Confirmed cases were defined as any suspected case with a positive culture for *V. cholerae* or with an epidemiologic link with a confirmed case. Not all suspected cases undergo confirmatory testing. As of January 3, 2023, MSPP had reported 280 institutional (health care facility) deaths and 177 community deaths through the daily alert-based reporting system^§^ (a system through which surveillance officers obtain daily counts of cases and deaths from the reporting facilities); the moving 14-day CFR was 3.0% (Figure 1). As of January 3, Haiti’s case-based surveillance system^¶^ had detected 20,262 suspected cases, 1,332 (7%) of which were culture-confirmed; 16,019 (79%) patients had been hospitalized. Among reported patients with suspected cholera, 11,580 (57%) are males, and the median age is 21 years (ranging from <1 to 100 years). Approximately one third (36%) of patients with suspected cholera are aged <10 years (Table). The highest proportion of suspected cholera cases (20%) and deaths (17%) occurred among children aged 1–4 years (4,009 and 48, respectively).

**TABLE:** Characteristics of cholera outbreak cases — Haiti, September 2022–January 2023*

Characteristic	No. (%)	
	Suspected cholera cases^†^	Cholera deaths
**Age group, yrs**		
<1	376 (1.9)	2 (0.7)
1–4	4,009 (19.8)	48 (16.8)
5–9	2,890 (14.3)	30 (10.5)
10–14	1,383 (6.8)	13 (4.6)
15–19	1,041 (5.1)	9 (3.2)
20–29	2,970 (14.7)	31 (10.9)
30–39	2,803 (13.8)	26 (9.1)
40–49	1,931 (9.5)	39 (13.7)
50–59	1,378 (6.8)	31 (10.9)
60–69	879 (4.3)	26 (9.1)
70–79	441 (2.2)	19 (6.7)
≥80	161 (0.8)	11 (3.9)
**Sex**		
Female	8,682 (42.8)	109 (38.2)
Male	11,580 (57.2)	176 (61.8)
**Department**		
Ouest	14,176 (70.0)	170 (59.6)
Artibonite	3,134 (15.5)	66 (23.2)
Centre	1,250 (6.2)	0 (—)
Nippes	517 (2.6)	7 (2.5)
Nord-Ouest	490 (2.4)	16 (5.6)
Grand’Anse	254 (1.3)	9 (3.2)
Sud-Est	185 (0.9)	10 (3.5)
Nord	155 (0.8)	2 (0.7)
Sud	95 (0.5)	5 (1.8)
Nord-Est	6 (<1.0)	0 (—)

* From case-based surveillance reporting.

^†^ Department was missing for two suspected cases.

**FIGURE 2:**
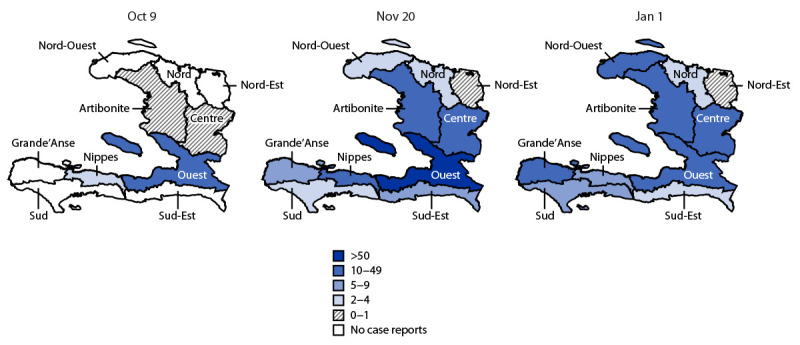
Rolling 14-day cholera incidence (cases per 100,000 population), by department — Haiti, October 2022–January 2023

The epicenter of the outbreak was the greater Port-au-Prince area, located in Ouest Department; as of January 3, based on Haiti’s case-based surveillance system, Port-au-Prince metropolitan area had reported 63% of all suspected cases (12,695) and 51% of all deaths (144) in the country. The peak in suspected cases occurred on November 8, after which case counts have steadily declined. However, *V. cholerae* transmission continues to occur throughout the country. As of January 3, MSPP had reported confirmed cases in nine of 10 departments (all except Nord-Est), and suspected cases have been reported in all 10 departments (Figure 2). The actual number of incident cases is likely substantially higher than that reported, given that incidence** to date has closely mirrored reporting from cholera treatment centers, and 79% of all patients with suspected cholera have been hospitalized. This indicates that occurrence of community-based surveillance is limited, which could hinder the ability to detect cholera cases that can propagate transmission.

## Public Health Response

CDC is working closely with MSPP, the U.S. Agency for International Development, and implementing partners (including MSF and the Pan American Health Organization) to further expand epidemiologic and laboratory surveillance to guide ongoing response needs. These include improving data collection and ensuring that a sufficient number of cholera treatments centers, beds for patient care, and oral rehydration points are available, to reduce morbidity and mortality. Support is also being provided to departmental health directorates to implement local investigations and response activities to contain cholera in areas of high transmission. Efforts are underway to improve access to WASH services and to support risk communication and community engagement nationwide to interrupt community transmission of cholera throughout Haiti. Support was also provided to launch an OCV campaign in mid-December in high-transmission areas.

## Discussion

The first cholera outbreak in Haiti was reported in October 2010, 10 months after the catastrophic earthquake that killed >200,000 persons and displaced >1 million. The 2010 outbreak resulted in >820,000 cases and approximately 10,000 deaths (*1*,*2*). Nearly 12 years after the outbreak began, Haiti was declared cholera-free on February 4, 2022, after 3 years without a confirmed case (*1*). Haiti is currently experiencing a resurgence of cholera that affects all parts of the country. The ongoing social unrest has negatively affected public health infrastructure, creating an environment that has facilitated the current resurgence and associated high mortality across the country. In addition, recent fuel shortages have hindered water treatment efforts and other cholera response activities nationwide. These factors have reduced the supply of safe drinking water, forcing an increasing number of residents to rely on unsafe sources and untreated water, substantially worsening the cholera outbreak and hindering the response (*5*,*6*).

Cholera outbreaks, especially in the setting of a complex humanitarian crisis, can spread rapidly, result in many deaths, and quickly become a public health crisis. Mild cases that are not seen in health care facilities can propagate transmission; thus, their detection is critical for monitoring and controlling transmission. Prompt and effective treatment for patients with cholera can reduce mortality rates from >50% to <1% (*6**,**7*). Primary treatment includes rehydration therapy (prompt restoration of lost fluids and salts); antibiotic treatment is recommended for severe cholera cases only (*8*). A CFR of <1% is the goal for case management interventions. Recent peaks in the 14-day CFR at the beginning of December and January might be elevated because of recent receipts of large numbers of backlogged death reports and because of safety and security concerns making community-based surveillance challenging, thereby limiting the detection of less severe cases of cholera. Efforts to control the outbreak and reduce CFRs should include a combination of surveillance, WASH services, risk communication and community engagement, timely treatment of illness, and OCVs. According to WHO’s position paper on cholera vaccines, OCVs should be used in humanitarian crises with high risk for cholera, during a cholera outbreak, and in places with endemic cholera, always in conjunction with other cholera prevention and control strategies (*9*).

The resurgence of cholera in Haiti and the complexity of the response present significant challenges. However, the existing technical capacity in Haiti, which was built during the previous cholera response, has provided valuable experience and staffing resources to combat cholera. Lessons learned about how to treat and prevent cholera from the previous response should be leveraged to aggressively respond to this outbreak and ensure effective public health actions. Although cases have declined, a multipronged approach including strengthened surveillance, timely case management, targeted OCV campaigns, risk communication, community engagement, and access to safe WASH services and emergency water treatment are urgently needed.

**Summary**

**What is already known about this topic?**

The first cholera outbreak in Haiti was reported in October 2010. Haiti was declared cholera-free in February 2022, after 3 years with no confirmed cases.

**What is added by this report?**

On October 2, 2022, two cases of *Vibrio cholerae* O1 infection were confirmed in the greater Port-au-Prince area. As of January 3, 2023, >20,000 suspected cholera cases had been reported throughout the country.

**What are the implications for public health practice?**

Multiple factors, including social unrest, have affected public health infrastructure and facilitated cholera resurgence. Although cases have declined, a multipronged approach, including sufficient and timely case management, strengthened surveillance, emergency water treatment, and targeted oral cholera vaccination campaigns are urgently needed.

## Footnotes

* Sect. 45 C.F.R. part 46.102(l)(2), 21 C.F.R. part 56; 42 U.S.C. Sect. 241(d); 5 U.S.C. Sect. 552a; 44 U.S.C. Sect. 3501 et seq.

† Haiti is divided into 10 departments, which are further divided into 42 arrondissements and 145 communes.

§ From alert-based reporting.

¶ From case-based surveillance reporting.

** Cases per 100,000 persons.
